# Subject-Based Model for Reconstructing Arterial Blood Pressure from Photoplethysmogram

**DOI:** 10.3390/bioengineering9080402

**Published:** 2022-08-18

**Authors:** Qunfeng Tang, Zhencheng Chen, Rabab Ward, Carlo Menon, Mohamed Elgendi

**Affiliations:** 1 School of Electronic Engineering and Automation, Guilin University of Electronic Technology, Guilin 541004, China; 2 Department of Electrical and Computer Engineering, University of British Columbia, Vancouver, BC V6T 1Z2, Canada; 3 Biomedical and Mobile Health Technology Laboratory, Department of Health Sciences and Technology, ETH Zurich, 8008 Zurich, Switzerland

**Keywords:** digital health, data science, intensive and critical care, cardiology, electrocadiogram, vital sign analysis, biosignal reconstruction, signal mapping

## Abstract

The continuous prediction of arterial blood pressure (ABP) waveforms via non-invasive methods is of great significance for the prevention and treatment of cardiovascular disease. Photoplethysmography (PPG) can be used to reconstruct ABP signals due to having the same excitation source and high signal similarity. The existing methods of reconstructing ABP signals from PPG only focus on the similarities between systolic, diastolic, and mean arterial pressures without evaluating their global similarity. This paper proposes a deep learning model with a W-Net architecture to reconstruct ABP signals from PPG. The W-Net consists of two concatenated U-Net architectures, the first acting as an encoder and the second as a decoder to reconstruct ABP from PPG. Five hundred records of different lengths were used for training and testing. The experimental results yielded high values for the similarity measures between the reconstructed ABP signals and their reference ABP signals: the Pearson correlation, root mean square error, and normalized dynamic time warping distance were 0.995, 2.236 mmHg, and 0.612 mmHg on average, respectively. The mean absolute errors of the SBP and DBP were 2.602 mmHg and 1.450 mmHg on average, respectively. Therefore, the model can reconstruct ABP signals that are highly similar to the reference ABP signals.

## 1. Introduction

Cardiovascular disease (CVD) is the leading cause of death worldwide, and its prevalence is increasing yearly [1]. Arterial blood pressure (ABP) is an essential indicator of the functioning of the cardiovascular system. An ABP that is too high or too low will affect the blood supply of all organs and increase the burden on the heart. If the ABP is too low, the blood supply to the organs will be reduced, especially to vital organs, such as the brain and heart, which could lead to severe consequences. At the same time, the heart and blood vessels are overburdened if the blood pressure is too high. In clinical practice, non-invasive cuff-based or the continuous invasive arterial catheters measure blood pressure depending on the patient’s situation. The cuff-based non-invasive blood pressure monitoring method has played a significant role in detecting hypertension and preventing cardiovascular diseases. However, only systolic and diastolic blood pressure values, and not waveforms, can be obtained. An ABP waveform contains further information about the heart rate, heart rate variability, and arrhythmia, besides the systolic and diastolic blood pressure. Furthermore, the morphological characteristics of ABP signals can be used to assess the cardiac output [2]. However, in continuous monitoring, invasive (intravascular) measurements have potential risks for patients, such as infection, site bleeding, and vascular damage. Therefore, non-invasive continuous blood pressure-monitoring has a significant clinical value.

**Figure 1:**
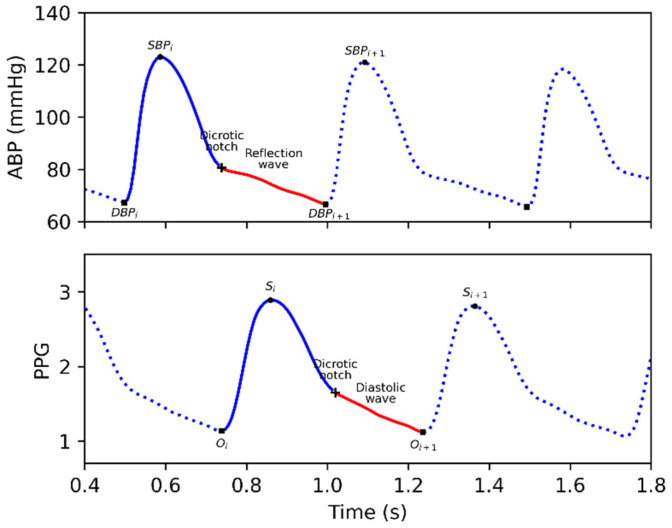
A synchronized ABP and PPG signal. *SBP_i_* and *DBP_i_* in ABP refer to the *i*th systolic and diastolic blood pressure, respectively. *O_i_* and *S_i_* stand for the *i*th onset and systolic peak, respectively. For the *i*th beat, the blue line is the systolic wave in ABP and PPG. This figure was used to show the correlation between ABP and PPG. Note that PPG = Photoplethysmogram, ABP = Arterial blood pressure, SBP = Systolic blood pressure, and DBP = Diastolic blood pressure.

The photoplethysmogram (PPG), another signal reflecting the state of the cardiovascular system, has attracted extensive attention in recent years due to its ease with respect to collection, its small sensor size, and its non-invasiveness. The PPG has been used to estimate oxygen saturation [3], blood pressure [4], cardiac output [5], and other physiological characteristics. Figure 1 shows a pair of synchronized ABP and PPG signals. It is easy to see that the ABP and PPG are highly similar in their waveforms. The two main feature points in an ABP cycle are the systolic blood pressure (SBP) and diastolic blood pressure (DBP). In a clinical setting, blood pressure classification is usually based on the values of these two feature points. There are also two main features of a PPG cycle: the onset and the systolic peak. However, because there is no uniform standard, the systolic peak and onset values in PPG are usually of no physiological significance. It should be noted that for the same heartbeat cycle, the delay in the systolic peak of the PPG relative to the SBP of ABP is mainly due to the difference in the measurement site. In terms of physiology, the pressure and volume of the arteries change periodically during each cardiac cycle, such as the heart’s systole and diastole phases. These changes propagate as pulse waves along the arterial wall toward the peripheral blood vessels. The PPG signal is the pulse wave signal of the blood volume changes in the peripheral blood vessels obtained by an optical method. Although ABP and PPG are measured at different sites and with different methods, they share the same excitation source of the heart. As shown in Figure 1, the blue part of the *i*th beat in the ABP, defined as a systolic wave, stands for the heart’s systole. While the red part, which was defined as a reflection wave, stands for the heart’s diastole [6,7]. The dicrotic notch demarcates the end of systole and the beginning of diastole [8,9]. Similar to the ABP signal, there is also a systolic wave, a dicrotic notch, and a diastolic wave in a PPG beat [10,11,12]. Furthermore, in signal analysis, the PPG and ABP are consistent in the time and frequency domains, and there is a significant causality from ABP to PPG [13]. Therefore, it is reasonable to use PPG to reconstruct the ABP signal.

Regarding the reconstruction of ABP signals using PPG signals, to our knowledge, there are only two ways to accomplish this. The PPG2ABP method takes segments of a long period of PPG and ABP as inputs and compares the performance of U-Net and Multi-ResUNet architectures in converting PPG segments into ABP waveform segments [14]. The other approach is the ABP-Net, which is an improved version of the Wave-U-NET architecture. The model’s input contains three signals: PPG, the PPG’s first derivative (VPG), and the PPG’s second derivative (APG) [15]. These two studies used SBP, DBP, and MAP to evaluate the model’s performance [14,15]. However, the ABP is a time series signal, and feature points cannot accurately evaluate the morphological similarity between the original ABP signal and the reconstructed ABP. Therefore, the use of some similarity measures of the waveform is needed in the performance evaluation.

In this study, we propose a W-Net deep neural network structure to reconstruct the ABP signal from PPG. In contrast to previous studies, to better evaluate the global similarity between the reconstructed ABP and the reference ABP, the Pearson’s correlation coefficient [16] and the dynamic time warping [17] measures are introduced for evaluating the model’s performance

## 2. Methodology

**Figure 2:**
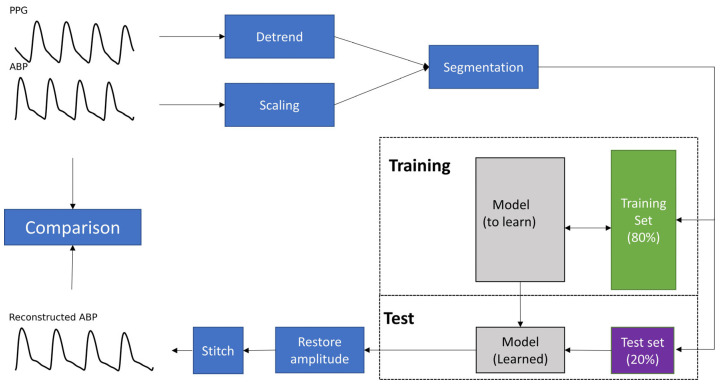
Flowchart of the proposed method. For each record, the PPG was detrended, and the ABP was scaled in preprocessing. Then, they were segmented into 8.2 s. The first 80% of segments were used for training, and the last 20% were used for testing. For the test set, the ABP signal of the segments was stitched to generate the reference ABP. In addition, the model’s outputs were stitched to generate the reconstructed ABP. Note that PPG = Photoplethysmogram and ABP = Arterial blood pressure.

The flowchart of the proposed method is shown in Figure 2. All steps will be described in detail in this section. This neural network was implemented in Tensorflow 2.8.0 for Python 3.9. All models were run in NVIDIA GeForce RTX 3060 Ti and Intel Core i7-11700 @ 2.50 GHz.

### 2.1. Dataset

**Figure 3:**
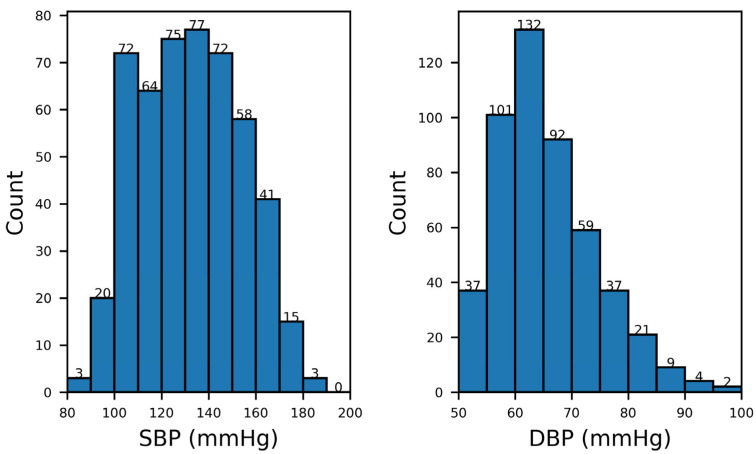
Histogram distributions of SBP and DBP of the data set. This figure shows that the dataset has a population with a large spectrum of blood pressure values. The used dataset can test the model’s performance under different blood pressures. Note that SBP = Systolic blood pressure and DBP = Diastolic blood pressure.

The data used in this article are from a cuffless blood pressure estimation data set [18]. This data set is one that Kachuee et al. compiled from the MIMIC II database [19]. It consists of a total of 12,000 records with varying lengths that contain synchronized PPG, ABP, and lead II ECG signals. All signals were sampled at 125 Hz. In this study, only PPG and ABP signals were used. Referring to previous works, we removed records less than 8 min in length [13,14]. In addition, records with a maximum value above 200 mmHg in the ABP signal were removed. After this removal, the number of records decreased to 2064. This paper uses only the first 500 of these 2064 records. Histogram distributions of the SBP and DBP of these records are shown in Figure 3. In this study, the SBP and DBP were extracted by an ABP delineator algorithm [20].

### 2.2. Preprocessing

- Detrending. Since the PPG can be easily corrupted by movement [21], it is necessary to remove the trend in PPG signals. In this study, the results of the linear least-squares fit to PPG were removed from PPG as trends.
- Scaling. The activation function of the last layer in the model architecture is tanh, so the amplitude of the output should range from [−1, 1]. For this paper, all ABP signals were divided by 200 to scale them in a range of [0, 1].
- Segmentation. After detrending and scaling, the PPG and ABP signals were divided into segments of 8.192 s (including 1024 samples). The overlap between the two consecutive segments was 75%. Due to the varying lengths of records in the dataset, the number of segments generated by this step for each record may differ.
- Split training and test set. The segments generated by the segmentation step were then split into training and test sets. The first 80% of the segments were defined as the training set, and the last 20% were defined as the test set to generate a continuous ABP signal.

### 2.3. Model Choice

**Figure 4:**
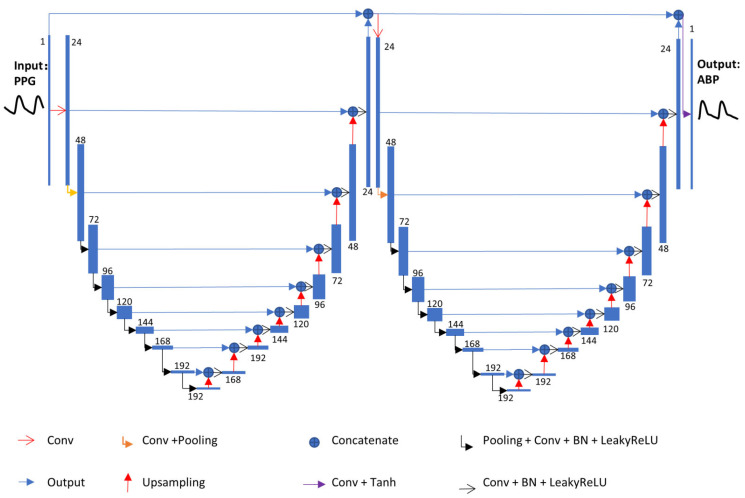
Architecture of the proposed network. The architecture consists of two concatenated U-Net architectures, the first acting as an encoder and the second as a decoder reconstructing ABP from PPG. Due to the shape of the whole architecture, this neural network was named W-Net.

As shown in Figure 4, the proposed network architecture consists of two U-Net blocks. The method comprising two U-blocks has been proven to obtain better performance than one U-block in the image analysis area [22,23].

“Conv” in Figure 4 denotes a one dimensional convolution layer. “Pooling” and “Upsampling” stand for the average pooling layer and the up-sampling in the time direction by 2, respectively. “Tanh” and “LeakyReLU” refer to the activation functions of the corresponding convolution layers. “BN” denotes a 1D batch normalization layer. The slope of the leakyReLU activation was set to 0.1.

### 2.4. Restoring Amplitude of the ABP

The signal values of the output by the model were in the range of [0, 1]. This is because the ABP in the training set had been scaled during the preprocessing step. This step multiplied the output signal by 200 to restore the actual amplitude.

### 2.5. Stitching the Reconstruction of ABP Segments

The model’s output consisted of ABP segments with a length of 1024 samples with 75% overlap between the two consecutive segments. Therefore, we needed to stitch the segments together to obtain a continuous predictive ABP signal. When the two segments were stitched together, the last 75% of the ABP of the first segment was discarded, and the second segment was placed behind the remaining segments. The stitched signal was then used as the first signal and the next segment was stitched as the second segment. This step was repeated until all segments of the record were stitched.

### 2.6. Training Options

The optimization method of this proposed model is the Adam optimizer. This model was trained for 500 epochs with a batch size of 128. The learning rate was set to 0.001 for an initial value and decayed by 0.1. The loss function used in this study was defined as follows:

(1) $$\left\{\begin{array}{l} mse=\frac{1}{l}\sum_{i=1}^{l}{\left(ABP_{ref}\left(i\right)-ABP_{rec}\left(i\right)\right)}^{2}\\ r=\frac{\sum_{i=1}^{l}\left(ABP_{ref}\left(i\right)-\bar{ABP_{ref}}\right)\sum_{i=1}^{l}\left(ABP_{rec}\left(i\right)-\bar{ABP_{rec}}\right)}{\sqrt{\sum_{i=1}^{l}{\left(ABP_{ref}\left(i\right)-\bar{ABP_{ref}}\right)}^{2}}\sqrt{\sum_{i=1}^{l}{\left(ABP_{rec}\left(i\right)-\bar{ABP_{rec}}\right)}^{2}}}\\ mal=\max_{1\leq i\leq l}\left(|ABP_{ref}\left(i\right)-ABP_{rec}\left(i\right)|\right)\\ Loss=0.05\times mal+mse+\left(1-|r|\right), \end{array}\right.$$

where *mse*, *r*, and *mal* are mean squared error, Pearson’s correlation coefficient (*r*), and maximal absolute loss (MAL), respectively. *ABP_ref_*(*i*) and *ABP_rec_*(*i*) are the individual sample points of the reference ABP and the reconstructed ABP indexed with *i*, respectively. The variable *l* is the sample size of the reference ABP. The variables $\bar{ABP_{ref}}$ and $\bar{ABP_{rec}}$ are the means of the sample values of the reference ABP and the reconstructed ABP, respectively.

The mean squared error and (*r*) were used to restrict the global similarity between the reconstructed ABP and the reference ABP. The *r* was applied to measure the linear correlation between the two variables [16]. The value of *r* was in the range of [−1, 1], where ±1 indicates the strongest possible agreement and 0 indicates the strongest possible disagreement. The MAL was used to punish the abnormal samples, which has already been proven to improve the ABP-Net’s performance [15]. Since the maximum ABP signal used in this study is 200 mmHg, the MAL was divided by 200 to scale it to [0, 1].

### 2.7. Performance Evaluation

Five measures were used to evaluate the performance of the reference ABP signal and the reconstructed ABP in the proposed model: root mean squared error (*rmse*), Pearson’s correlation coefficient (*r*), normalized dynamic time warping (DTW) distance, and mean absolute error of SBP and DBP.

#### 2.7.1. Root Mean Square Error (*rmse*)

In machine learning, *rmse* is commonly used to measure the model’s estimated and observed values. The formula for *rmse* is as follows:

(2) $$rmse=\sqrt{\frac{1}{l}\sum_{i=1}^{l}{\left(ABP_{ref}\left(i\right)-ABP_{rec}\left(i\right)\right)}^{2}}.$$

#### 2.7.2. Mean Absolute Error (*MAE*) of SBP and DBP

SBP and DBP are the main features of the ABP signal, so their accuracy is important when evaluating the model’s performance. The ABP delineator may have errors when extracting SBP and DBP. To decrease the error in calculating the *MAE*, we found the SBP in the reconstructed ABP that corresponded to the SBP in the reference signal. That is, for each SBP extracted from the reference ABP, we found one of the SBPs of the reconstructed ABP that differed from its position by less than 10 sampling points and then defined it as the corresponding SBP. If it was not found, this SBP point in the reference ABP was discarded. Similarly, the DBP in the reconstructed ABP also corresponded to the DBP in the reference ABP. Then, the *MAE* between the corresponding SBP and DBP was calculated as a measure of similarity. The *MAE* of the SBP and DBP was defined as follows:

(3) $$MAE_{SBP}=\frac{1}{n}\sum_{i=1}^{l}|SBP_{ref}\left(i\right)-SBP_{rec}\left(i\right)|,$$

(4) $$MAE_{DBP}=\frac{1}{n}\sum_{i=1}^{l}|DBP_{ref}\left(i\right)-DBP_{rec}\left(i\right)|,$$

where *SBP_ref_*(*i*) and *SBP_rec_*(*i*) are the *i*th subjects of the average of the reconstructed and reference SBPs, respectively. *DBP_ref_*(*i*) and *DBP_rec_*(*i*) are the *i*th subject of the average of the reconstructed and reference DBPs, respectively. The *n* stands for the number of SBPs and DBPs.

#### 2.7.3. Normalized DTW Distance

To calculate the normalized DTW distance, it was first necessary to obtain the DTW distance. DTW can be used to measure the similarity between two time-series with potentially different velocities [17]. The reconstructed ABP signals might have had a small offset from the reference ABP, and their similarity under the best matching conditions could be assessed using DTW. The steps to calculate DTW were as follows:

- Create a *N×N* matrix. An element in the *i*th row and the *j^th^* column in the matrix is the Euclidean distance between the *i*th sample point in the reconstruction ABP and *j^th^* sample point in the reference ABP, which is defined as *d_i j_*.
- Look for the optimal path to minimize the sum of *d*_11_ to *d_NN_* along this path. This path is defined as the warping path, and the sum is the DTW distance.

The smaller the DTW distance, the more similar the two time series were. However, it can easily be seen that the DTW distance increased as the time series continued. To better evaluate the similarity of the two time series, in this study, the DTW distance was divided by the sum of the lengths of the reference ABP and the reconstructed ABP for normalization. The formula used was as follows:

(5) $$\bar{d}=\frac{d}{2N^{,}}$$

where *d* is the DTW distance, $\bar{d}$ is the normalized DTW distance, and *N* is the length of the reference ABP signal. The *rmse*, *r*, and $\bar{d}$ were used to measure the global similarity between the reconstructed and reference ABPs. The *MAE* of SBP and DBP were used to evaluate the main feature similarity.

## 3. Results

To better evaluate the model’s performance, three methods were used in this study.

- Method I. Using the W-Net architecture, the model’s input was only the PPG.
- Method II. Using the W-Net architecture, three signals were used for the inputs: the PPG, the velocity of the PPG (VPG), and the acceleration of the PPG (APG). The VPG and APG are the first and second deviations of the PPG signals, respectively. The ABP-Net shows that using VPG and APG as the inputs can improve the performance of the model. In this case, it was necessary to compare the model’s performance with and without the VPG and APG signals as inputs. In this study, the deviation step was defined as follows:

(6) $$X^{’}\left(i\right)=\left\{\begin{array}{l} X\left(2\right)-X\left(1\right),\;i=1\\ \frac{1}{2}\left(X\left(i+1\right)-X\left(i-1\right)\right),1\leq i\leq N-1\\ X\left(N\right)-X\left(N-1\right),i=N \end{array}\right.,$$

where *X^′^*(*i*) and *X*(*i*) denote the *i*th sample point of the raw and deviation signal, respectively, and *N* is the length of the PPGs. Note that the VPG and APG were generated before the segmentation and were segmented as the PPG simultaneously. The input demotion of the model was 3 × 1024 when trained with the VPG and APG.

- Method III. Using the left half of the W-Net architecture, which was the same as ABP-Net, the model inputs were the PPG, VPG, and APG. This method was used to compare W-Net’s performance with that of the ABP-Net.

Table 1 shows the results of the three methods. The average of the *rmse* for all the records in Method III was 4.873 mmHg. Whereas the averages of the *rmse* for all the records in Methods I and II were 2.236 mmHg and 2.234 mmHg, respectively. Both were less than half of that in method III. For the other measures, the results of Methods I and II were better than those of Method III. In this case, the performance of the W-Net architecture was better than that of the U-Net.

For Methods I and II, Pearson’s *r* was on average 0.995, which means the reconstructed ABPs were highly correlated with the reference ABPs. For the *rmse* and normalized DTW distances, the results of Methods I and II were very close.

**Figure 5:**
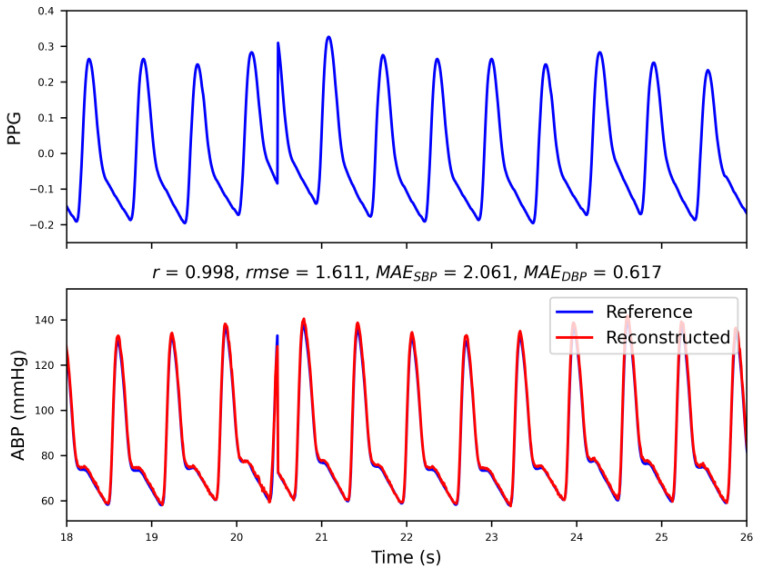
Demonstration of the reconstruction of the ABP waveform, where ‘*r*’ and ‘*rmse*’ stand for the correlation coefficient and the relative mean squared error, respectively, and ‘*MAE_SBP_*’ and ‘*MAE_DBP_*’ represent the mean absolute error of systolic and diastolic blood pressure, respectively. Note that PPG = Photoplethysmogram, ABP = Arterial blood pressure, SBP = Systolic blood pressure, and DBP = Diastolic blood pressure.

Figure 5 shows a demonstration of the reconstructed ABP waveform in Method I. The PPG had a delay relative to the ABP. The model can learn this delay during training. The Pearson’s r between the reconstructed ABP and reference ABP was 0.998. In this case, the proposed model was well matched with the waveform of the reconstructed ABP that matched the reference one. It should be noted that an arrhythmia occurs at 20–21 s, and the predicted ABP can match these events.

**Figure 6:**
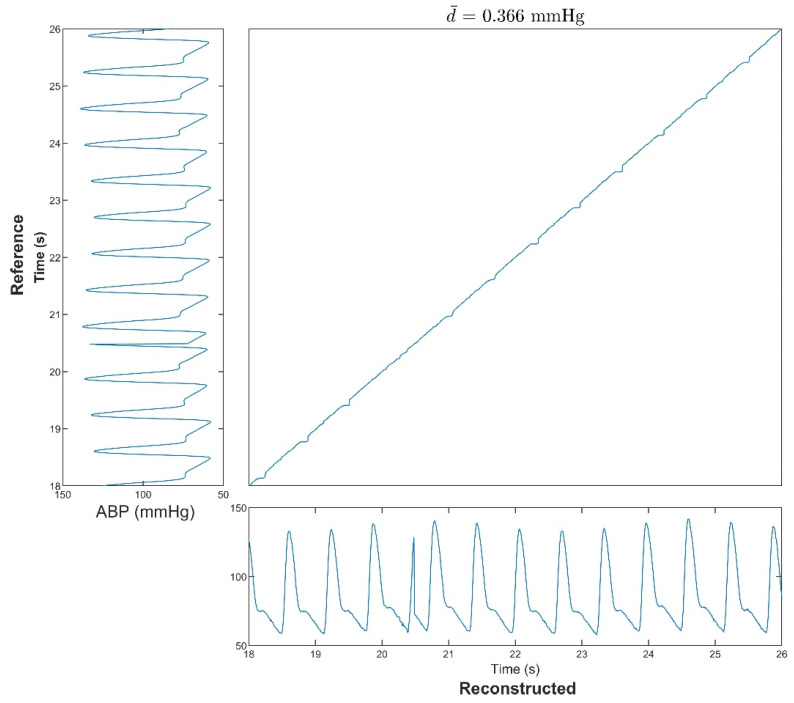
A segment of the optimal DTW warping path for the reference and reconstructed ABPs. The $\bar{d}$ stands for the normalized DTW distance. This figure shows that the reconstructed ABP was highly similar to the reference ABP. Note that ABP = Arterial blood pressure and DTW = Dynamic time warping.

To better evaluate the similarity between the reconstructed and reference ABPs in the nonlinear domain, the DTW was introduced. Figure 6 shows a segment of the optimal DTW warping path. This segment has the same time window as Figure 5. The normalized distance of this subject was 0.366 mmHg along the optimal warping path, which resembles a straight line. This means that a small amount of warping was required to obtain the optimal path, and there was a high degree of similarity between the two signals.

## 4. Discussion and Limitations

In recent years, numerous studies have shown that PPG signals can be used to assess blood pressure values. These methods can be classified into two main categories. The first involves obtaining some handcraft features (e.g., pulse wave velocity [24], pulse transit time [25], and pulse arrival time [26]) from a single or multiple signals to estimate the values of SBP, DBP, and mean arterial pressure (MAP). The other is the end-to-end prediction of blood pressure based on deep learning technology [27,28]. An ABP signal contains SBP, DBP, and other features such as a dicrotic notch and reflected wave. These features also include information about the cardiovascular system. Therefore, reconstructing the complete ABP waveform can provide more information about the cardiovascular system.

We proposed the use of the W-Net architecture to reconstruct ABP waveforms from PPG signals. Table 2 shows a comparison between this method and those from other research on topic-specific models. Compared to the other two models, our main advantage is that our model can evaluate the global similarity between the reconstructed ABP and the reference ABP. The similarity between systolic and diastolic blood pressure is crucial for the model. However, for a time series, the similarity of local feature points does not represent global similarity. Therefore, we introduced other metrics. Pearson’s *r* and rmse were mainly used to evaluate the linear similarity between the reconstructed ABP and the reference ABP. However, the pulses of PPG are later than those of ABP. Accordingly, deep learning models need to learn this delay to obtain more similar signals. To evaluate similarity in the presence of a delay, we introduced the normalized DTW distance. The DTW can find the distance under the best match between two time series to better assess similarity. The *MAE* of this paper is superior to those of the other two studies for both SBP and DBP [13,14]. However, the *MAE* of the SBP and DBP were affected by the accuracy of the feature extraction algorithm, which is one of the reasons why we introduced global similarity. It is challenging to extract a dicrotic notch due to its morphology varying alongside the patient-specific underlying physiological and pathological conditions. This paper did not check the consistency of the dicrotic notches and the reflection waves between the reconstructed and reference ABP. However, the average Pearson’s *r* for 500 records reached 0.995. A high value of the Pearson’s r indicates that the dicrotic notch and reflection wave in the reconstructed ABP were highly similar to the reference ABP.

A limitation of this paper is that we focus only on subject-specific models. One reason for this is that different circulatory diseases may alter the shape of the ABP and PPG waveforms. Another reason is that the time delay between PPG and ABP may differ for different subjects. Applying the model to multiple subjects means that the model needs to learn to distinguish between different shapes and delays in PPG and ABP, which is a challenge for our model. In future work, we will continue to improve the model so that it can be applied to multiple subjects. Another limitation of this study is that the algorithm was developed to reconstruct an ECG without considering the extreme blood pressure fluctuations. Blood pressure waveforms contain rhythmic or non-rhythmic fluctuations, such as respiration, vasodilation, and contraction, reflecting the cardiovascular control mechanisms [29]. A consideration of the fluctuations in ABP signals can provide a broader description of cardiovascular regulation. One of our future works will involve checking the consistency between the fluctuation in the reconstructed and the reference ABP signal. The dataset we used in this study was compiled from the MIMIC II dataset. However, the subject information has been removed, and we cannot obtain disease information for the subject. Therefore, the proposed model was not tested against different disease conditions. On the other hand, although the data set contains various types of ABP and PPG signals in a morphology associated with a large spectrum of blood pressure values, the subjects’ health conditions were provided. Therefore, the model’s performance on PPG signals collected from subjects with different arrhythmia conditions needs to be examined in future work. Multiple publicly-available PPG datasets could be used to overcome age and gender biases [30,31].

## 5. Conclusions

This paper proposed a deep neural network with a W-Net architecture to reconstruct ABP signals using PPG. A Pearson correlation was added to the loss function and used to evaluate the similarity between the reconstructed ABP signals and the reference signal. The experimental results show that this model can reconstruct ABP signals that are highly similar to the reference signals. Furthermore, compared with other models, the differences between the key features of SBP, DBP, and the reference ABP and the reconstructed ABP signal are minor. In the future, we will consider generalizing the model by applying it to multiple people to improve its applicability.

## Figures and Tables

**Table 1:** Comparison of the model’s performance in different settings. The *rmse* and *r* stand for the root mean squared error and Pearson’s correlation coefficient, respectively, and *MAE_SBP_* and *MAE_DBP_* stand for the mean absolute error between the systolic and diastolic blood pressure, respectively. The bold indicates the best performance in each evaluation metric. Note that PPG = Photoplethysmogram, VPG = Velocity PPG, APG = Acceleration PPG, SBP = Systolic blood pressure, DBP = Diastolic blood pressure, and DTW = Dynamic time warping.

	Net Architecture	Inputs	*rmse* (mmHg)	*MAE_SBP_* (mmHg)	*MAE_DBP_* (mmHg)	Pearson’s *r*	Normalized DTW Distance ($\bar{d}$)
Methods I	W-net	PPG	2.236 *±* 1.551	**2.602 *±* 1.886**	**1.450 *±* 1.330**	0.995 *±* 0.014	**0.612 *±* 0.270**
Methods II	W-net	PPG + VPG + APG	**2.234 *±* 1.523**	2.627 *±* 2.035	1.567 *±* 1.432	**0.995 *±* 0.013**	0.616 *±* 0.269
Methods III	U-net	PPG + VPG + APG	4.873 *±* 2.357	3.248 *±* 2.246	2.187 *±* 1.859	0.974 *±* 0.029	0.889 *±* 0.403

**Table 2:** Comparison of the performance of W-Net with those for existing methods in subject-specific model. The ’NR’ stands for not reported. Note that PPG = Photoplethysmogram, ABP = Arterial blood pressure, SBP = Systolic blood pressure, DBP = Diastolic blood pressure, and DTW = Dynamic time warping.

	Inputs	*MAE_SBP_*	*MAE_DBP_*	*rmse*	Pearson’s *r*	Normalized DTW Distance ($\bar{d}$)
This study	PPG	2.602 ± 1.886	1.450 ± 1.330	2.236 ± 1.551	0.995 ± 0.014	0.612 ± 0.270
	PPG + VPG + APG	2.627 ± 2.035	1.567 ± 1.432	2.234 ± 1.523	0.995 ± 0.013	0.616 ± 0.269
PPG2ABP	PPG	5.73 ± 9.16	3.45 ± NR	NR	NR	NR
ABP-Net	PPG + VPG + APG	3.27 ± 3.92	1.90 ± 2.44	3.20 ± NR	NR	NR

## Data Availability

The data used in this manuscript can be downloaded from this link https://archive.ics.uci.edu/ml/datasets/Cuff-Less+Blood+Pressure+Estimation (accessed on 2 April 2022).

## References

[1] Thomas H., Diamond J., Vieco A., Chaudhuri S., Shinnar E., Cromer S., Perel P., Mensah G.A., Narula J., Johnson C.O. (2018). Global Atlas of Cardiovascular Disease 2000–2016: The Path to Prevention and Control. Glob. Heart.

[2] Arya V., Kobe J., Mishra N., Al-Moustadi W., Nates W., Kumar B. (2019). Cardiac Output Monitoring: Technology and Choice. Ann. Card. Anaesth..

[3] Shelley K.H. (2007). Photoplethysmography: Beyond the Calculation of Arterial Oxygen Saturation and Heart Rate. Anesth. Analg..

[4] Elgendi M., Fletcher R., Liang Y., Howard N., Lovell N.H., Abbott D., Lim K., Ward R. (2019). The Use of Photoplethysmography for Assessing Hypertension. NPJ Digit. Med..

[5] Wang L., Pickwell-Macpherson E., Liang Y.P., Zhang Y.T. Noninvasive Cardiac Output Estimation Using a Novel photoplethysmogram Index. Proceedings of the 2009 Annual International Conference of the IEEE Engineering in Medicine and Biology Society.

[6] Polónia J., Barbosa L., Silva J.A., Rosas M. (2009). Improvement of Aortic Reflection Wave Responses 6 Months After Stopping Smoking: A Prospective Study. Blood Press. Monit..

[7] Sheng-Chi K., Chang C.C., Hsiao T.C., Hsu H.Y. Reflection Wave Analysis Based on Ensemble Empirical Mode Decomposition. Proceedings of the 2013 E-Health and Bioengineering Conference (EHB).

[8] Janney J.B., Umashankar G., Krishnakumar S., Chandana H., Chriselda L.C. (2021). Recognition of Dicrotic Notch in Arterial Blood Pressure Pulses Using Signal Processing Techniques. J. Phys. Conf. Ser..

[9] Saffarpour M., Basu D., Radaei F., Vali K., Adams J.Y., Chuah C.N., Ghiasi S. Dicrotic Notch Identification: A Generalizable Hybrid Approach under Arterial Blood Pressure (ABP) Curve Deformations. Proceedings of the 2021 43rd Annual International Conference of the IEEE Engineering in Medicine & Biology Society (EMBC).

[10] Elgendi M. (2012). On the Analysis of Fingertip Photoplethysmogram Signals. Curr. Cardiol. Rev..

[11] He X., Goubran R.A., Liu X.P. (2014). Secondary Peak Detection of PPG Signal for Continuous Cuffless Arterial Blood Pressure Measurement. IEEE Trans. Instrum. Meas..

[12] Elgendi M. (2020). PPG Signal Analysis: An Introduction Using MATLAB^®^.

[13] Martínez G., Howard N., Abbott D., Lim K., Ward R., Elgendi M. (2018). Can Photoplethysmography Replace Arterial Blood Pressure in the Assessment of Blood Pressure?. J. Clin. Med..

[14] Ibtehaz N., Rahman M.S. (2020). Ppg2abp: Translating Photoplethysmogram (PPG) Signals to Arterial Blood Pressure (ABP) Waveforms Using Fully Convolutional Neural Networks. arXiv.

[15] Cheng J., Xu Y., Song R., Liu Y., Li C., Chen X. (2021). Prediction of Arterial Blood Pressure Waveforms from Photoplethysmogram Signals via Fully Convolutional Neural Networks. Comput. Biol. Med..

[16] Liu J., Tang W., Chen G., Lu Y., Feng C., Tu X.M. (2016). Correlation and Agreement: Overview and Dlarification of Competing Concepts and Measures. Shanghai Arch. Psychiatry.

[17] Efrat A., Fan Q., Venkatasubramanian S. (2007). Curve Matching, Time Warping, and Light Fields: New Algorithms for Computing Similarity between Curves. J. Math. Imaging Vis..

[18] Kachuee M., Kiani M.M., Mohammadzade H., Shabany M. Cuff-Less High-Accuracy Calibration-Free Blood Pressure Estimation Using Pulse Transit Time. Proceedings of the 2015 IEEE International Symposium on Circuits and Systems (ISCAS).

[19] Saeed M., Villarroel M., Reisner A.T., Clifford G., Lehman L.W., Moody G., Heldt T., Kyaw T.H., Moody B., Mark R.G. (2011). Multiparameter Intelligent Monitoring in Intensive Care II: A Public-access Intensive Care Unit Database. Crit. Care Med..

[20] Li B.N., Dong M.C., Vai M.I. (2010). On An Automatic Delineator for Arterial Blood Pressure Waveforms. Biomed. Signal Process. Control.

[21] Kim B., Yoo S. (2006). Motion Artifact Reduction in Photoplethysmography Using Independent Component Analysis. IEEE Trans. Biomed. Eng..

[22] Gare G.R., Li J., Joshi R., Magar R., Vaze M.P., Yousefpour M., Rodriguez R.L., Galeotti J.M. (2021). W-Net: Dense and Diagnostic Semantic Segmentation of Subcutaneous and Breast Tissue in Ultrasound Images by Incorporating Ultrasound RF Waveform Data. Med. Image Anal..

[23] Gargiulo M., Dell’Aglio D.A.G., Iodice A., Riccio D., Ruello G. (2020). Integration of Sentinel-1 and Sentinel-2 Data for Land Cover Mapping Using W-Net. Sensors.

[24] Koivistoinen T., Lyytikäinen L.-P., Aatola H., Luukkaala T., Juonala M., Viikari J., Lehtimäki T., Raitakari O.T., Kähönen M., Hutri-Kähönen N. (2018). Pulse Wave Velocity Predicts the Progression of Blood Pressure and Development of Hypertension in Young Adults. Hypertension.

[25] Ding X., Yan B.P., Zhang Y.T., Liu J., Zhao N., Tsang H.K. (2017). Pulse Transit Time Based Continuous Cuffless Blood Pressure Estimation: A New Extension and A Comprehensive Evaluation. Sci. Rep..

[26] Liang Y., Abbott D., Howard N., Lim K., Ward R., Elgendi M. (2019). How Effective Is Pulse Arrival Time for Evaluating Blood Pressure? Challenges and Recommendations from a Study Using the MIMIC Database. J. Clin. Med..

[27] Hsu Y.-C., Li Y.-H., Chang C.-C., Harfiya L.N. (2020). Generalized Deep Neural Network Model for Cuffless Blood Pressure Estimation with Photoplethysmogram Signal Only. Sensors.

[28] Harfiya L., Chang C.-C., Li Y.-H. (2021). Continuous Blood Pressure Estimation Using Exclusively Photopletysmography by LSTM-Based Signal-to-Signal Translation. Sensors.

[29] Parati G., Saul J.P., Di Rienzo M., Mancia G. (1995). Spectral Analysis of Blood Pressure and Heart Rate Variability in Evaluating Cardiovascular Regulation: A Critical Appraisal. Hypertension.

[30] Elgendi M., Fletcher R.R., Tomar H., Allen J., Ward R., Menon C. (2021). The Striking Need for Age Diverse Pulse Oximeter Databases. Front. Med..

[31] Sinaki F.Y., Ward R., Abbott D., Allen J., Fletcher R.R., Menon C., Elgendi M. (2022). Ethnic Disparities in Publicly-available Pulse Oximetry databases. Commun. Med..

